# Role of inflammatory biomarkers in mediating the effect of lipids on spontaneous intracerebral hemorrhage: a two-step, two-sample Mendelian randomization study

**DOI:** 10.3389/fneur.2024.1411555

**Published:** 2024-08-07

**Authors:** Mingsheng Huang, Yiheng Liu, Yuan Cheng, Weiran Dai

**Affiliations:** ^1^Department of Neurosurgery, The Second Affiliated Hospital of Chongqing Medical University, Chongqing, China; ^2^Department of Cardiology, The Second Affiliated Hospital of Chongqing Medical University, Chongqing, China

**Keywords:** lipids, inflammatory factors, sICH, Mendelian randomization, causal effect

## Abstract

**Background:**

Spontaneous intracerebral hemorrhage (sICH) is a form of stroke with high mortality rates and significant neurological implications for patients. Abnormalities in lipid metabolism have been implicated in various cardiovascular diseases, yet their relationship with sICH remains insufficiently explored, particularly concerning their association with inflammatory factors.

**Methods:**

Employing a two-sample, two-step Mendelian Randomization approach, combined with data from GWAS datasets, to investigate the causal relationship between plasma lipid levels and sICH. Additionally, the role of inflammatory factors in this relationship was examined, and sensitivity analyses were conducted to ensure the robustness of the results.

**Results:**

The results indicate a significant causal relationship between 19 plasma lipid metabolites and sICH. Furthermore, mediation analysis revealed that three distinct lipids, namely Sterol ester (27:1/20:2), Phosphatidylcholine (16:0_20:4), and Sphingomyelin (d34:1), exert their influence on sICH through inflammatory factors. TRAIL (OR: 1.078, 95% CI: 1.016–1.144, *p* = 0.013) and HGF (OR: 1.131, 95% CI: 1.001–1.279, *p* = 0.049) were identified as significant mediators.

**Conclusion:**

This study provides new evidence linking abnormalities in lipid metabolism with sICH and elucidates the role of inflammatory factors as mediators. These findings contribute to a better understanding of the pathogenesis of sICH and offer novel insights and therapeutic strategies for its prevention and treatment.

## Introduction

Spontaneous intracerebral hemorrhage (sICH), resulting from the rupture of blood vessels in the brain, accounts for 10–15% of total stroke occurrences and represents a significant contributor to neurological morbidity and mortality, with a 30-day mortality rate ranging from 35 to 52%. Half of these fatalities occur within the initial 48 h, imposing a significant burden on families and society (1, 2). The incidence of sICH increases with age, particularly among individuals aged 65 and above. However, it can also occur in younger populations, especially in the presence of risk factors. Hypertension is one of the primary risk factors for sICH. Other risk factors include smoking, alcohol consumption, vascular diseases, diabetes, and hyperlipidemia (3–5).

Lipids constitute a fundamental category of organic compounds present in living organisms, encompassing fats, fatty acids, triglycerides, phospholipids, cholesterol, and other constituents. Lipids are indispensable for various cellular functions, including cell membrane integrity, energy storage and release, signaling cascades, and hormone synthesis (6, 7). Dyslipidemia, characterized by aberrant lipid metabolism, is intricately linked to the development and progression of numerous ailments, such as cardiovascular diseases, obesity, fatty liver disease, and metabolic syndrome (8, 9). In terms of the impact of dyslipidemia on cerebrovascular ailments, prior investigations have established that conditions like hypercholesterolemia and atherosclerosis can result in cerebral blood vessel constriction or obstruction, with hyperlipidemia emerging as a recognized risk factor for ischemic stroke (10). While previous studies have primarily focused on the relationship between lipids and ischemic stroke, emerging evidence suggests that dyslipidemia may also be associated with the risk of sICH (11–13). When lipid metabolism becomes abnormal, a series of complex biological changes may occur in the body, directly or indirectly increasing the risk of hemorrhagic stroke. Firstly, abnormal lipid metabolism may recruit immune cells and lead to the release of pro-inflammatory factors. These inflammatory responses can result in endothelial cell damage, leading to structural changes in the blood vessel walls (14, 15). Endothelial damage may constitute a fundamental basis for increased vessel susceptibility to rupture (15). Secondly, abnormal lipid metabolism may accelerate the formation and development of atherosclerotic plaques. The instability of these plaques increases the risk of rupture, particularly in complex plaques where macrophage-driven inflammatory responses and macrophage apoptosis may lead to the release of lipid contents, thereby further promoting plaque rupture (14, 16). Therefore, abnormal lipid metabolism may lead to structural changes in the blood vessel walls, reduced elasticity, and increased brittleness, thereby making the vessels more prone to rupture and bleeding. Previous studies have found that low total cholesterol levels may promote cell necrosis in the arterial intima, rendering it susceptible to microaneurysms and associated with the onset of hemorrhagic stroke (17).

Moreover, certain lipid substances, such as low-density lipoprotein cholesterol, may promote platelet activation and tissue factor expression, leading to impaired coagulation function, which may play a role in the pathogenesis of cerebral hemorrhage (18). These mechanisms may interact with each other, collectively increasing the risk of hemorrhagic stroke occurrence. In conclusion, the etiology of sICH is multifactorial, with inflammation potentially augmenting vascular permeability and exacerbating vascular pathologies such as arteriosclerosis and aneurysm formation, thereby increasing the risk of vascular rupture and subsequent intracranial bleeding (14). This finding has spurred further investigation into the role of lipid substances in the pathophysiology of stroke, offering new perspectives for the development of novel therapeutic strategies. Therefore, exploring the relationship between lipid substances and sICH holds significant importance.

Mendelian randomization (MR) is an analytical approach that leverages genetic variation to mimic randomized controlled trials (RCTs), facilitating causal inference regarding the relationship between risk factors and diseases (19). It serves to mitigate the influence of confounding variables and address issues of reverse causation. It has been extensively employed to investigate causal associations between exposures and diseases (19, 20). Based on the aforementioned conditions and background, a two-sample MR analysis will be conducted to elucidate the relationship between lipid levels and sICH. Subsequently, a mediation MR analysis will be performed to assess the potential role of inflammatory factors within this association, thereby exploring the underlying mechanistic pathways involved.

## Materials and methods

### Data resources for plasma lipidome, inflammatory biomarkers, and sICH

Data of plasma lipidome were obtained from a univariate and multivariate GWAS involving 179 lipid species across 13 lipid classes from 7,174 Finnish individuals in the GeneRISK cohort (21). This study performed a phenome-wide association study on identified lipid-related genetic loci among 377,277 participants from the FinnGen biobank, followed by colocalization analysis of these endpoints (accession numbers GCST90277238 to GCST90277287).

The inflammatory biomarkers data were obtained from Zhao et al.’s study (22). In this research, a thorough evaluation of genetic influences on inflammation-related proteins was conducted using a genome-wide analysis of protein quantitative trait loci. The study encompassed 14,824 participants of European descent and involved the measurement of 91 plasma proteins via the Olink panel. Accession numbers GCST90274758 to GCST90274848.

The data of sICH were acquired from the FinnGen dataset consisting of 7,040 cases and 374,631 controls of European ancestry.^1^ The FinnGen project is an open large-scale genetic research initiative originating from Finland, utilizing samples from extensive populations across various regions within the country. Its objective is to employ genetic research methodologies, particularly genome-wide association studies (GWAS), to elucidate the associations between genetic variations and diseases as well as health characteristics. The FinnGen dataset comprises substantial genomic, clinical, and biosample information, which can be utilized for investigating a multitude of diseases, including but not limited to cardiovascular diseases, tumors, metabolic disorders, and neurological conditions (23).

### Selection of genetic instrumental variables

According to previous researches (24, 25), we employed a threshold of *p* < 1 × 10^-5 to select SNPs associated with the plasma lipidome. In the reverse MR analysis, SNPs associated with sICH were selected using the same criteria. Subsequently, we performed a clumping procedure to ensure independence among the chosen SNPs (*r*^2 < 0.001, clumping window = 10,000 kb) (26). To assess the reliability of each SNP, we computed the *F*-statistic and retained only those with values exceeding 10 (27). This stringent criterion ensured the robustness of our instrumental variables. The *F*-statistic for each SNP was calculated using the formula *F* = R^2 / (1 − *R*^2) × (*N*-2), where *R*^2 represents the variance of exposure explained by the instrumental variables (IVs), and N denotes the sample size. Furthermore, we calculated the variance of exposure explained by the instrument variable using the formula *R*^2 = *β*^2 / (*β*^2 + se^2 × *N*), where *β* denotes the effect size for the genetic variant of interest, she represents the standard error for *β*, and *N* indicates the sample size.

### Statistical analysis

We utilized the Inverse Variance Weighting (IVW) method as our primary approach, which effectively leverages multiple single nucleotide polymorphisms (SNPs) as instrumental variables to estimate the causal impact of the exposure on the outcome. This method aggregates effect estimates of individual SNPs through inverse variance weighting, aiming to maximize the precision of the combined effect and thereby yield robust (28). Additionally, we employed weighted mode, MR-Egger, Simple mode, and weighted median tests to assess the effects, ensuring accuracy and robustness by detecting causal relationships from various aspects.

**Figure 1:**
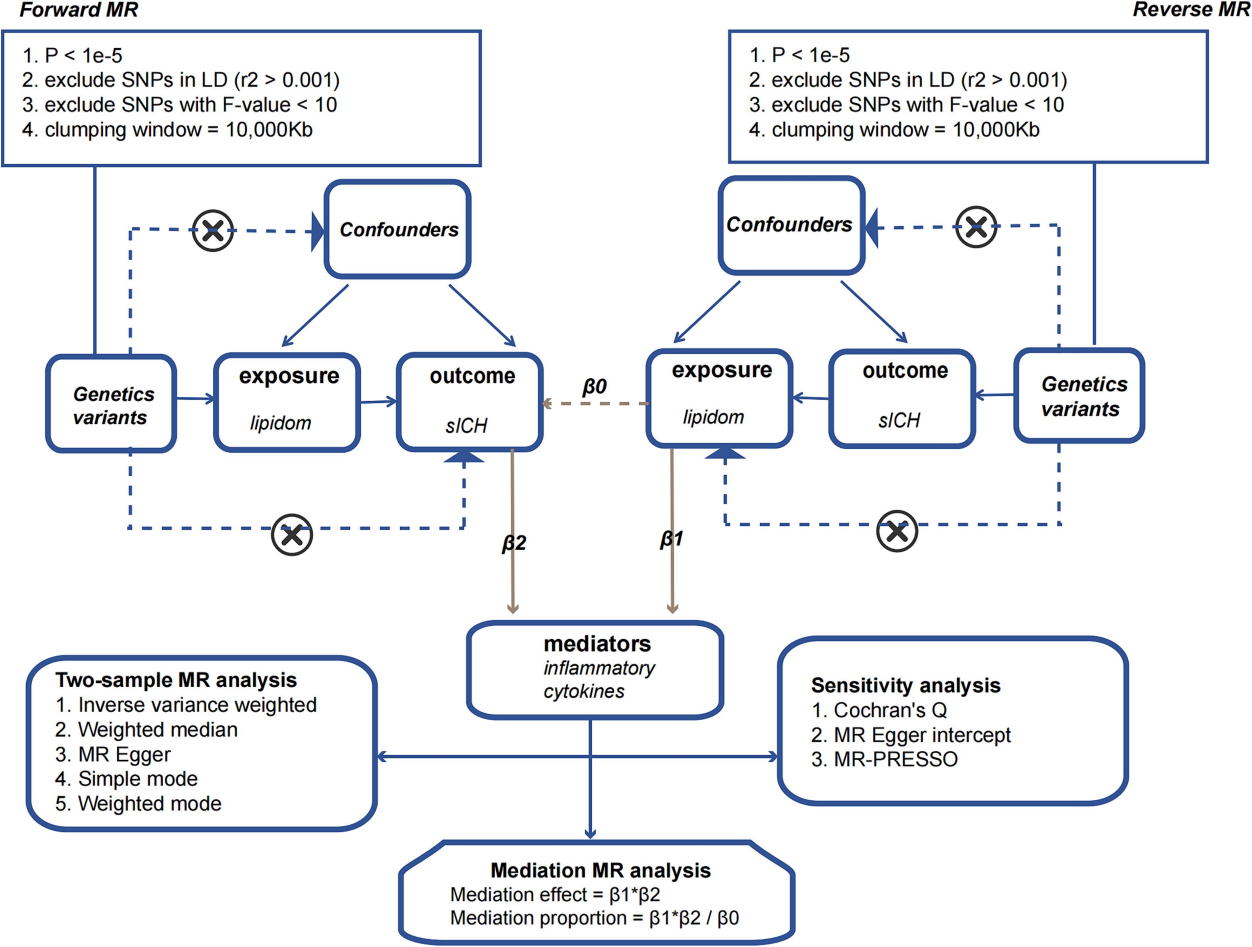
The flowchart illustrating the Mendelian randomization process.

We employed a two-sample MR approach to dissect the direct and indirect effects of the plasma lipidome on sICH. In addition to the fundamental effect estimate (β1) of the plasma lipidome on inflammatory factors obtained from univariable MR analysis, two additional estimates were calculated: the causal effect of the mediator (inflammatory biomarkers) on sICH (β2), and the total effect of the plasma lipidome on sICH (β0). The mediation effect refers to the causal impact of the plasma lipidome on sICH through the mediator (inflammatory biomarkers), which can be estimated using the coefficient product method (β1 × β2). Hence, the proportion of the indirect effect can be calculated as “mediation effect/total effect” ([β1 × β2]/β0) (Figure 1).

### Sensitivity analysis

Sensitivity analysis involves assessing the robustness of causal estimates to potential biases and confounding factors. We utilized Cochran’s Q test to evaluate the heterogeneity in the impact of genetic variants on the exposure factor. Additionally, MR-Egger intercept and MR-PRESSO analyses were conducted to assess the potential influence of pleiotropy on the outcomes of the MR analysis. Simultaneous utilization of scatter plots and funnel plots for data visualization and quality assurance aided in a comprehensive examination of the accuracy and reliability of the analytical results. Finally, a leave-one-out sensitivity analysis was conducted, removing one SNP at a time, to investigate if any single SNP was responsible for the causal association.

The effect estimates were reported as odds ratios (ORs) with their 95% confidence intervals (CIs). Statistical significance was defined as a two-sided *p*-value <0.05. All analyses were conducted using the “TwoSampleMR” package (version 0.5.8) in the R software (version 4.3.1).

## Results

### Effect of plasma lipidome on spontaneous intracerebral hemorrhage

Assessed primarily through the IVW method, the findings indicate significant causal associations between genetically predicted levels of 19 distinct plasma lipidome components and the risk of sICH. These components include Sterol ester (27:1/16:0) (OR: 0.938, 95% CI: 0.883–0.996, *p* = 0.036), Sterol ester (27:1/20:2) (OR: 1.088, 95% CI: 1.016–1.166, *p* = 0.016), Sterol ester (27:1/20:4) (OR: 0.925, 95% CI: 0.886–0.967, *p* = 0.000), Phosphatidylethanolamine (16:0_18:2) (OR: 1.066, 95% CI: 1.011–1.124, *p* = 0.019), Phosphatidylinositol (16:0_18:1) (OR: 1.158, 95% CI: 1.052–1.276, *p* = 0.003), and Sphingomyelin (d34:1) (OR: 0.929, 95% CI: 0.871–0.992, *p* = 0.027) (Table 1, causal effects assessed using different methods are presented in Supplementary Table S1). In subsequent reverse MR analyses, employing the IVW method, we confirmed a causal relationship between the plasma levels of Sterol ester (27:1/20:4) (OR: 0.936, 95% CI: 0.883–0.991, *p* = 0.024) and Phosphatidylcholine (18:0_20:4) (OR: 0.931, 95% CI: 0.879–0.986, *p* = 0.015) with the occurrence of sICH. The remaining 17 lipid species showed no reverse causal relationship with sICH (Table 2, detailed data in Supplementary Table S2).

**Table 1:** The causal effects of plasma lipidom on sICH by IVW method.

Plasma lipidom	nsnp	Beta	OR	or_lci95	or_uci95	*p*-value
Sterol ester (27:1/16:0)	34	−0.064	0.938	0.883	0.996	0.036
Sterol ester (27:1/20:2)	27	0.085	1.088	1.016	1.166	0.016
Sterol ester (27:1/20:4)	31	−0.078	0.925	0.886	0.967	0.000
Sterol ester (27:1/22:6)	23	−0.132	0.876	0.800	0.960	0.004
Phosphatidylethanolamine (18:2_0:0)	27	0.084	1.088	1.024	1.156	0.007
Phosphatidylcholine (14:0_18:2)	26	0.098	1.103	1.001	1.215	0.047
Phosphatidylcholine (15:0_18:2)	39	0.060	1.062	1.003	1.123	0.037
Phosphatidylcholine (16:0_20:4)	24	−0.062	0.940	0.897	0.986	0.011
Phosphatidylcholine (16:0_20:5)	24	−0.074	0.929	0.863	1.000	0.049
Phosphatidylcholine (16:0_22:5)	29	−0.059	0.943	0.891	0.998	0.043
Phosphatidylcholine (17:0_20:4)	30	−0.078	0.925	0.877	0.975	0.003
Phosphatidylcholine (18:0_20:4)	27	−0.077	0.926	0.881	0.973	0.002
Phosphatidylcholine (18:1_18:3)	18	0.120	1.128	1.007	1.263	0.037
Phosphatidylcholine (O-17:0_17:1)	33	0.096	1.101	1.019	1.189	0.015
Phosphatidylethanolamine (16:0_18:2)	21	0.064	1.066	1.011	1.124	0.019
Phosphatidylinositol (16:0_18:1)	18	0.147	1.158	1.052	1.276	0.003
Sphingomyelin (d34:1)	32	−0.074	0.929	0.871	0.992	0.027
Sphingomyelin (d36:2)	24	−0.080	0.923	0.854	0.998	0.043
Triacylglycerol (52:3)	30	−0.075	0.928	0.862	0.999	0.047

IVW, Inverse variance weighting; sICH, Spontaneous intracerebral hemorrhage.

**Table 2:** The causal effects of sICH on plasma lipidom by IVW method.

Plasma lipidom	nsnp	Beta	OR	or_lci95	or_uci95	*p*-value
Sterol ester (27:1/16:0)	45	−0.037	0.964	0.909	1.021	0.213
Sterol ester (27:1/20:2)	45	−0.038	0.962	0.898	1.032	0.282
Sterol ester (27:1/20:4)	45	−0.067	0.936	0.883	0.991	0.024
Sterol ester (27:1/22:6)	45	−0.019	0.981	0.926	1.040	0.519
Phosphatidylethanolamine (18:2_0:0)	45	0.052	1.053	0.992	1.117	0.089
Phosphatidylcholine (14:0_18:2)	45	0.003	1.003	0.945	1.065	0.927
Phosphatidylcholine (15:0_18:2)	45	−0.014	0.986	0.917	1.060	0.700
Phosphatidylcholine (16:0_20:4)	45	−0.050	0.951	0.898	1.008	0.090
Phosphatidylcholine (16:0_20:5)	45	−0.007	0.993	0.934	1.056	0.831
Phosphatidylcholine (16:0_22:5)	45	−0.006	0.994	0.938	1.053	0.840
Phosphatidylcholine (17:0_20:4)	45	−0.027	0.974	0.918	1.032	0.368
Phosphatidylcholine (18:0_20:4)	45	−0.071	0.931	0.879	0.986	0.015
Phosphatidylcholine (18:1_18:3)	45	0.018	1.018	0.956	1.085	0.573
Phosphatidylcholine (O-17:0_17:1)	45	0.036	1.036	0.972	1.105	0.275
Phosphatidylethanolamine (16:0_18:2)	45	0.009	1.009	0.944	1.078	0.796
Phosphatidylinositol (16:0_18:1)	45	0.033	1.034	0.971	1.101	0.299
Sphingomyelin (d34:1)	45	−0.026	0.974	0.919	1.032	0.374
Sphingomyelin (d36:2)	45	−0.049	0.952	0.899	1.009	0.101
Triacylglycerol (52:3)	45	−0.015	0.985	0.918	1.056	0.670

IVW, Inverse variance weighting; sICH, Spontaneous intracerebral hemorrhage.

### Effect of plasma lipidome on inflammatory biomarkers

The impact of 17 different lipid substances on 91 inflammatory biomarkers was assessed. Causal relationships were found between 3 lipid species and 2 inflammatory biomarkers (Table 3; Supplementary Table S3). Sterol ester (27:1/20:2) (OR: 0.913, 95% CI: 0.913–0.996, *p* = 0.033) and Phosphatidylcholine (16:0_20:4) (OR: 0.897, 95% CI: 0.868–0.928, *p* = 0.000) were both negatively correlated with TNF-related apoptosis-inducing ligand levels (TRAIL). Additionally, plasma levels of Sphingomyelin (d34:1) were negatively correlated with Hepatocyte growth factor levels (HGF) (OR: 0.940, 95% CI: 0.890–0.992, *p* = 0.025).

**Table 3:** The causal effects of plasma lipidom on inflammatory biomarkers by IVW method.

Plasma lipidom	Inflammatory cytokines	nsnp	Beta	OR	95%CI	*p*-value
Sterol ester(27:1/20:2)	TRAIL	27	−0.047	0.954	0.913–0.996	0.033
Phosphatidylcholine(16:0_20:4)	TRAIL	24	−0.108	0.897	0.868–0.928	0.000
Sphingomyelin(d34:1)	HGF	32	−0.062	0.940	0.890–0.992	0.025

IVW, Inverse variance weighting; TRAIL, TNF-related apoptosis-inducing ligand levels; HGF, Hepatocyte growth factor levels.

### Effect of inflammatory biomarkers on spontaneous intracerebral hemorrhage

Increased levels of TRAIL (OR: 1.078, 95% CI: 1.016–1.144, *p* = 0.013) and HGF (OR: 1.131, 95% CI: 1.001–1.279, *p* = 0.049) were both associated with a higher risk of sICH (Table 4; Supplementary Table S4).

**Table 4:** The causal effects of inflammatory biomarkers on sICH by IVW method.

Inflammatory cytokines	nsnp	Beta	OR	or_lci95	or_uci95	*p*-value
TNF-related apoptosis-inducing ligand	36	0.075	1.078	1.016	1.144	0.013
Hepatocyte growth factor	30	0.123	1.131	1.001	1.279	0.049

IVW, Inverse variance weighting; sICH, Spontaneous intracerebral hemorrhage.

### Mediation effect of inflammatory biomarkers

**Figure 2:**
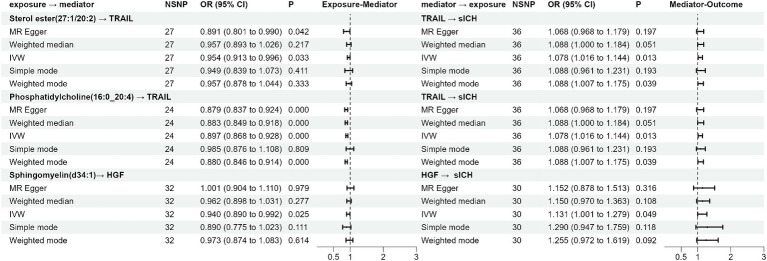
The forest plot of the Mendelian randomization analysis results.

TRAIL and HGF were significantly associated with both specific plasma lipidome and sICH. A mediation effect of plasma lipidome on sICH via TRAIL and HGF was observed. We found that the highest proportion was for the effect of plasma Phosphatidylcholine (16:0_20:4) concentration mediated by TRAIL on sICH, with a mediation effect of 13.2%, while the lowest was for the effect of plasma Sterol ester (27:1/20:2) concentration mediated by TRAIL on sICH, which was only 4.2%. The mediation effects of different mediators are demonstrated in Table 5. All the results of two-step MR analysis were demonstrated in Figure 2.

**Table 5:** The mediation effect and proportion of inflammatory biomarkers by IVW method.

Plasma lipidom	Inflammatory cytokines	β0	β1	β2	β1*β2	Mediation proportion (%)
Sterol ester(27:1/20:2)	TRAIL	0.085	−0.047	0.075	−0.004	4.2
Phosphatidylcholine(16:0_20:4)	TRAIL	−0.062	−0.108	0.075	−0.008	13.2
Sphingomyelin(d34:1)	HGF	−0.074	−0.062	0.123	−0.008	10.4

IVW, Inverse variance weighting; TRAIL, TNF-related apoptosis-inducing ligand levels; HGF, Hepatocyte growth factor levels.

### Sensitivity analysis

After rigorous screening, the number of eligible SNPs serving as IVs in the plasma lipidome for sICH at exposure are as follows: 27, 24, and 32, corresponding to Sterol ester (27:1/20:2), Phosphatidylcholine (16:0_20:4), and Sphingomyelin (d34:1). In the reverse MR analysis, based on our screening criteria, a total of 45 usable SNPs related to sICH were identified. When selecting SNPs to study the causal relationship between plasma lipidome as exposure and inflammatory factors as outcome, the determined range of available instrumental variables is from 24 to 32. Subsequently, In the process of screening for TRAIL and HGF instrumental variables, we found 36 and 30 variables, respectively. All SNPs had *F*-statistics ranging from 19.552 to 475.334. An F-statistic >10 is considered indicative of adequate instrument strength.

**Figure 3:**
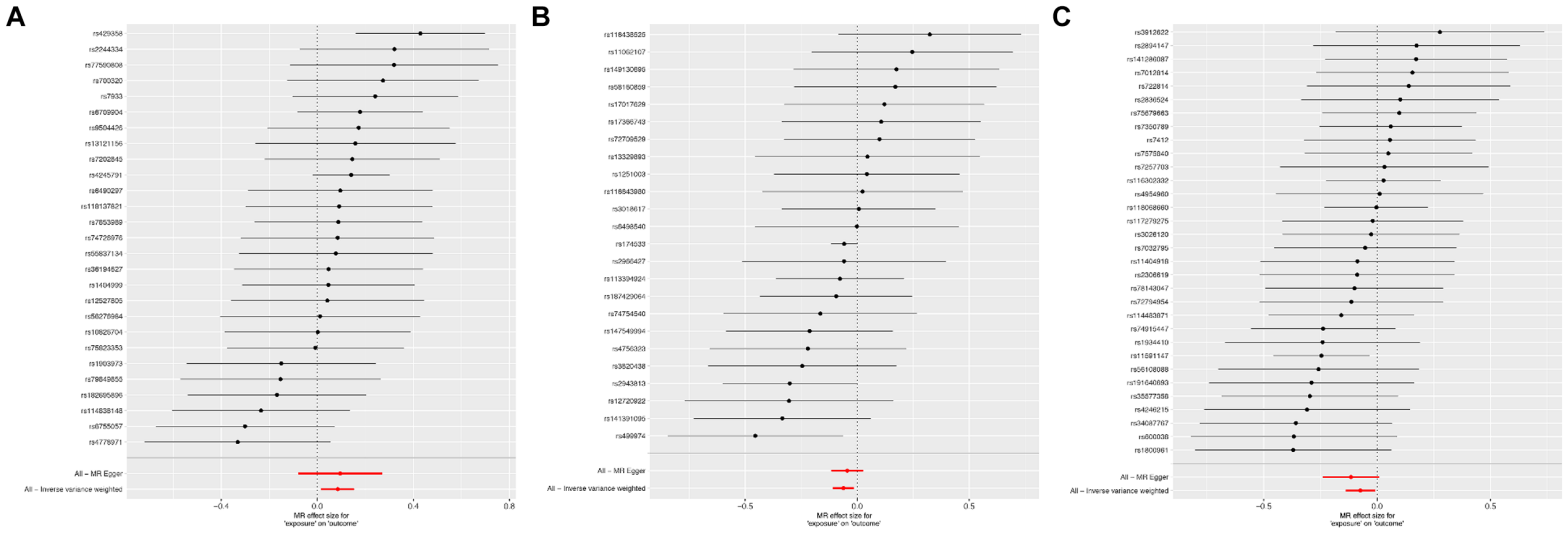
The forest plot from Sterol ester (27:1/20:2) **(A)**, Phosphatidylcholine (16:0_20:4) **(B)**, and Sphingomyelin (d34:1) **(C)** to spontaneous intracerebral hemorrhage.

**Figure 4:**
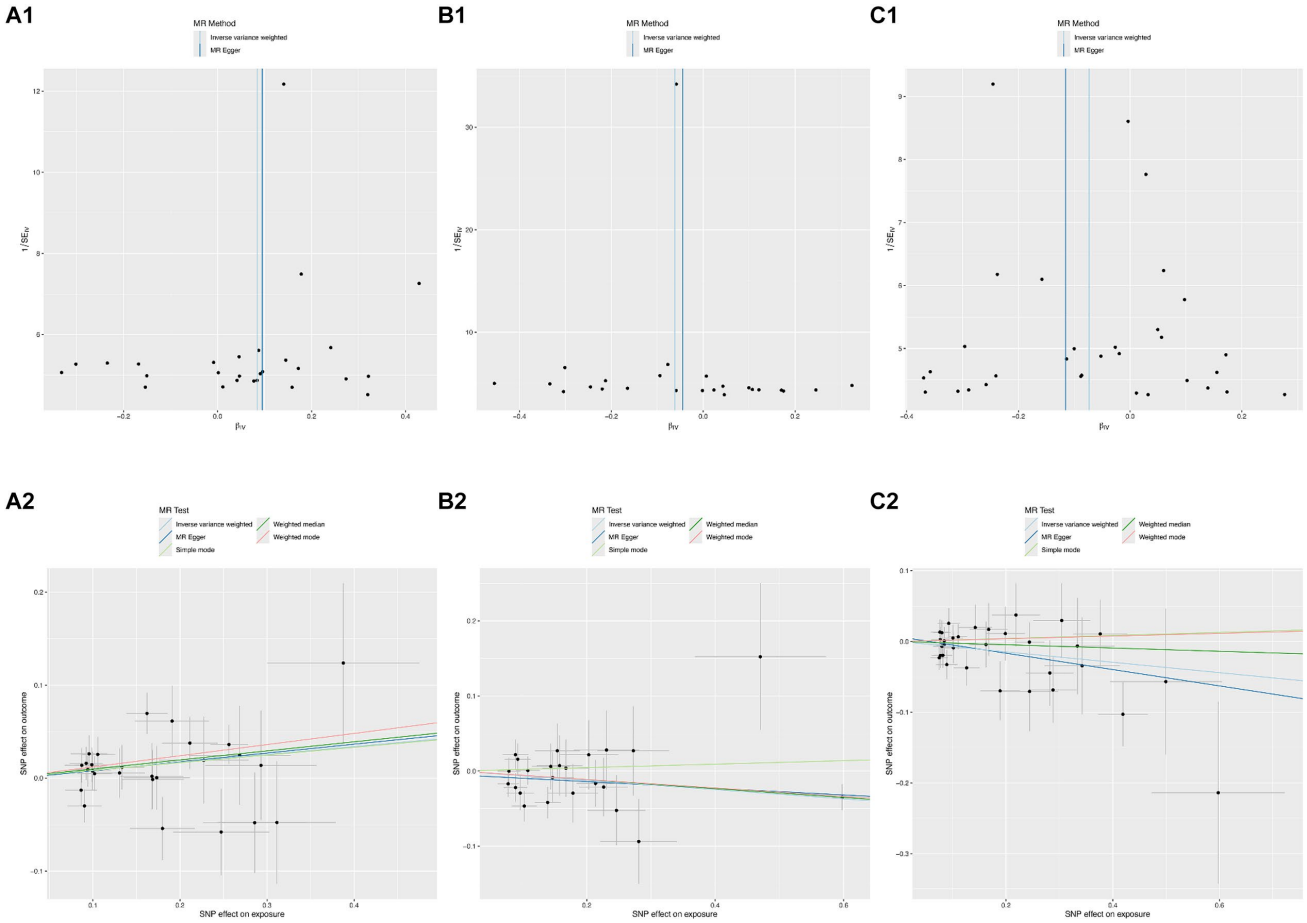
The funnel plots and scatter plots from Sterol ester (27:1/20:2) **(A1,A2)**, Phosphatidylcholine (16:0_20:4) **(B1,B2)**, and Sphingomyelin (d34:1) **(C1,C2)** to spontaneous intracerebral hemorrhage.

**Figure 5:**
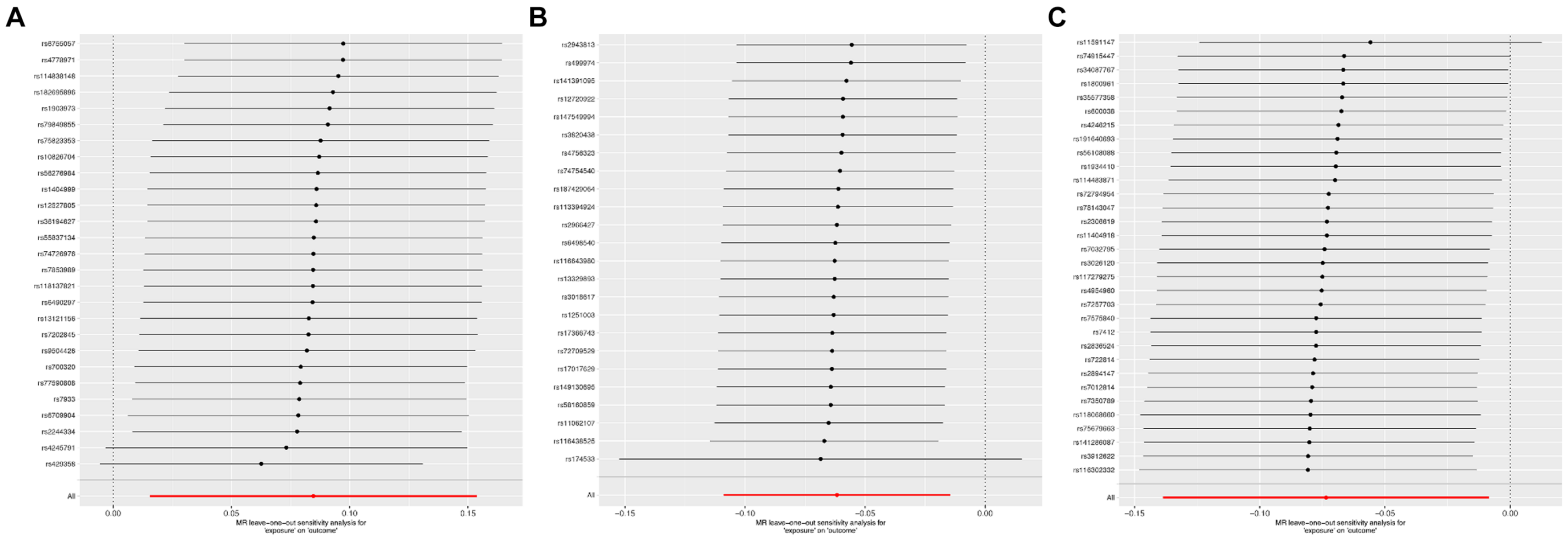
The leave-one-out sensitivity analysis from Sterol ester (27:1/20:2) **(A)**, Phosphatidylcholine (16:0_20:4) **(B)**, and Sphingomyelin (d34:1) **(C)** to spontaneous intracerebral hemorrhage.

According to Cochran’s Q test, there was no evidence of heterogeneity in the instrumental variables from the plasma lipidome to sICH. To assess the potential horizontal pleiotropy of SNPs, we employed MR-Egger regression, providing a valuable assessment of its presence. Sensitivity analysis results did not reveal significant evidence of directional pleiotropy (*p* > 0.05). During sensitivity analysis of the association between plasma lipidome and inflammatory factors, we observed no heterogeneity or horizontal pleiotropy in plasma lipidome traits. Furthermore, leave-one-out analysis demonstrated that no SNP significantly influenced the results (Figure 3), and the Scatter Plot and Funnel Plot revealed no apparent outliers or biases (Figure 4). The forest plot demonstrates that the effect estimates all point in the same direction, with no significant heterogeneity observed (Figure 5). By the research procedure, the relevant data are presented sequentially from Supplementary Tables S5–S7.

## Discussion

According to the current literature, this is the first study to investigate how inflammatory factors mediate the causal pathway between plasma lipidome levels and sICH. This research discovered a possible correlation between genetically determined plasma lipidome levels and the risk of sICH. Furthermore, additional mediation analysis supported that the causal effects of plasma lipidome levels on sICH were partially mediated by inflammatory factors.

Lipids are essential for cellular function, serving as fundamental constituents of cell membranes, contributing to energy storage, maintaining equilibrium, and regulating cellular signaling pathways (7). Lipids are integral constituents of the brain, and their imbalance is correlated with disorders of the nervous system. Perturbations in lipid metabolism are connected with vascular inflammation and oxidative stress, both critical factors contributing to the development of atherosclerosis (29). We identified Sterol ester (27:1/20:2), Phosphatidylcholine (16:0_20:4), and Sphingomyelin (d34:1) as belonging to the categories of lipid esters, phospholipids, and sphingolipids, respectively. Through inflammatory mediators, they are causally associated with the onset of sICH. Sterol esters, resulting from the esterification of sterols with fatty acids, are pivotal in preserving the structural and operational integrity of cellular membranes. They modulate membrane fluidity and stability, thereby impacting cellular responsiveness to external stimuli and signal transduction. Notably, within the nervous system, sterol esters assume a critical role, particularly in the formation and sustenance of myelin sheaths. These sheaths serve as protective coatings for nerve fibers, and the presence of sterol esters aids in safeguarding nerve fibers while facilitating efficient nerve signal transmission (30). Additionally, in the context of inflammation, sterol esters may serve as signaling molecules or regulatory factors. By modulating cellular signal transduction pathways, they can affect the expression of genes related to inflammation and the release of inflammatory mediators. This modulation helps regulate the polarization state of inflammatory cells and the balance between pro-inflammatory and anti-inflammatory factors, thereby influencing the onset and progression of inflammation (31). Phosphatidylcholine constitutes a fundamental constituent within cell membranes, comprising a glycerol scaffold, dual fatty acid chains, and a phosphoric acid moiety connecting a choline unit. It serves as a critical player in upholding membrane architecture, cellular signaling cascades, choline provisioning, and transportation, alongside the pathophysiological pathways involved in disease onset (32). Among these, choline is an important nutrient involved in the synthesis of the neurotransmitter acetylcholine, maintenance of cellular membrane integrity, and metabolism of methyl (33). The metabolic pathways of choline have been associated with ischemic stroke and cognitive dysfunction after acute ischemic stroke (34, 35). Sphingomyelin is a phospholipid compound found within cell membranes, exerting a crucial role in upholding membrane structure, cellular signaling, neurological system function, and metabolic modulation (36). Within the nervous system, it serves as a primary constituent of both neuronal cell membranes and myelin sheaths, essential for maintaining the structural integrity and functionality of neurons (37). Specifically, sphingomyelin contributes to the protection of neuronal cell membranes from external environmental damage while promoting the formation and maintenance of myelin sheaths, thereby ensuring efficient neural signal transmission (37). Moreover, studies suggest that Sphingomyelin acts as a signaling molecule in modulating the initiation and progression of inflammatory responses. Upon cellular inflammation stimulation, phospholipase catalyzes the hydrolysis of Sphingomyelin into phosphoric acid and choline, with phosphoric acid playing a pivotal role as a constituent of inflammatory signals. Phosphatidic acid can activate inflammatory signaling pathways, such as NF-κB and MAPK pathways, thereby promoting the production and release of inflammatory cytokines, leading to the initiation of inflammatory responses (38). In conclusion, three of them play crucial roles in cell membrane structure and function, serve as key players in the nervous system, and are involved in modulating inflammatory responses.

TRAIL, categorized as a surface protein, is a member of the tumor necrosis factor (TNF) family and is predominantly expressed by activated immune cells, including T and B lymphocytes, neutrophils, dendritic cells, monocytes, macrophages, natural killer cells, and Natural Killer T cells (NKT) (39). Its main role involves maintaining the internal balance of the immune system, responding to infections, autoimmune diseases, and apoptosis. TRAIL exhibits pro-angiogenic activity and stimulates the proliferation of vascular smooth muscle cells (40–42). Conversely, TRAIL has also been found to inhibit vascular endothelial growth factor (VEGF)-mediated angiogenesis through both caspase-8-dependent and caspase-8-independent mechanisms (43), thus demonstrating a dual functionality. TRAIL might impact vascular development by controlling the survival and apoptosis of endothelial cells and vascular smooth muscle cells. Recent studies have indicated that CD4+ T cells derived from atherosclerotic plaques induce apoptosis of Vascular Smooth Muscle Cells (VSMCs) through a TRAIL-dependent mechanism, potentially leading to plaque instability and rupture (44). Hence, apoptosis of vascular cells triggered by TRAIL may regulate cellular turnover within the vascular wall. TRAIL has the potential to trigger the activation of the NF-κB signaling pathway, resulting in heightened activation and transcriptional potency of NF-κB (45, 46). NF-κB can modulate the expression of inflammation-related genes in both endothelial cells and vascular smooth muscle cells, promoting the onset of inflammatory responses. Activation of inflammation within endothelial cells can lead to increased vascular permeability and leukocyte adhesion, thereby contributing to the development of inflammatory vascular diseases such as atherosclerosis. Additionally, during the process of angiogenesis, NF-κB influences the formation of new blood vessels by regulating the expression of angiogenesis-related factors such as VEGF and matrix metalloproteinases (MMPs) (47, 48). Although research on the relationship between TRAIL and sICH is currently lacking, making it challenging to ascertain the precise mechanisms involved, it is hypothesized that TRAIL might affect sICH by influencing the function and integrity of the vascular wall. Hepatocyte Growth Factor, also known as Hepatotropin, is a multifunctional protein. It is a cytokine produced by fibroblasts and other cells. HGF primarily exerts its biological functions through binding to its receptor, the c-Met receptor. This binding activates the c-Met receptor, triggering a cascade of signaling pathways such as Ras-MAPK, PI3K-Akt, and STAT pathways. Consequently, these signaling pathways regulate diverse biological processes such as cell proliferation, anti-fibrotic and anti-inflammatory responses, as well as tissue repair and regeneration (49–51). In the central nervous system, HGF is recognized as a neuroprotective factor, exerting a beneficial influence on neuronal survival and repair. In the context of ischemic stroke, HGF mitigates the decline in tight junction protein levels and diminishes Blood–Brain Barrier disruption, thereby ameliorating cerebral edema following ischemia. Moreover, it reduces neuroinflammatory responses and attenuates neurotoxic damage, thus aiding in the regulation of cerebral inflammatory reactions (52–55). Furthermore, HGF is implicated in modulating angiogenesis and neuroregeneration processes. It facilitates endothelial cell migration and proliferation, regulates vascular smooth muscle cell function, and amplifies the expression and functionality of additional angiogenic factors like VEGF, thereby expediting angiogenesis, which is vital for vascular formation and restructuring. Additionally, it fosters brain tissue restoration and reshaping by augmenting neuroregeneration mechanisms (56, 57). However, current research on the role of HGF in sICH is insufficient, making it difficult to determine whether it retains its protective effects in this subtype of stroke. It is even possible that HGF may increase the risk of sICH. Further animal and clinical studies are needed to verify these potential effects. Although our study identified that plasma levels of Sterol ester (27:1/20:2), Phosphatidylcholine (16:0_20:4) are associated with a reduced risk of sICH through TRAIL, and plasma Sphingomyelin (d34:1) levels are associated with a reduced risk of sICH through HGF, their effects are limited, requiring further investigation to explore the potential molecular mechanisms involved. Additionally, it is noteworthy that the correlation analysis for Sterol ester (27:1/20:2) presents a paradox. While its overall effect increases the risk of sICH, it can also reduce the likelihood of sICH by lowering TRAIL levels. The mechanisms involved may include dysregulated lipid metabolism not only increasing the expression of pro-inflammatory mediators but also altering factors such as immune cells and oxidative stress. These combined factors ultimately lead to an overall effect of increased sICH risk.

### Strengths and limitations

Our study utilized MR to infer causal relationships between exposure factors and diseases by leveraging genetic variation. Compared to observational studies, MR effectively reduces confounding factors, reverse causation, and information bias. Additionally, MR offers high statistical efficiency and minimizes reverse causation effects and information bias, making it a powerful tool for elucidating disease mechanisms. Despite these advantages, several limitations need to be addressed. MR results may be influenced by potential heterogeneity and genetic pleiotropy. Our study primarily focused on individuals of European descent, which may limit the generalizability of the findings, necessitating further investigation in diverse populations. Furthermore, although the initial threshold for exposure-related SNPs was set at *p* < 5×10^−8^, it was adjusted to *p* < 1×10^−5^ due to the limited availability of effective SNPs, potentially introducing some instability in the results. Future research should encompass populations of different ethnicities and geographical regions to validate the universality and reliability of our findings. While we identified inflammatory factors as potential mediators, we did not explore other biological pathways related to sICH mediated by lipid factors. Future studies should include additional mediators, such as immune cells and oxidative stress, and validate our findings across various populations. Further research should also investigate how inflammatory factors and lipid metabolism contribute to sICH. Integrating large-scale cohort data with multi-omics approaches could provide a more comprehensive understanding of the underlying mechanisms. Exploring potential intervention methods, including pharmacological treatments, lifestyle changes, and dietary adjustments, could help reduce sICH risk and improve outcomes.

## Conclusion

Through a two-step, two-sample MR analysis, this study provides robust evidence of a causal relationship between blood lipid levels and the risk of sICH. Additionally, the findings indicate that blood lipid levels may influence the risk of sICH via inflammatory pathways, thereby contributing novel insights for future strategies in prevention and monitoring.

## Data availability statement

The original contributions presented in the study are included in the article/Supplementary material, further inquiries can be directed to the corresponding author.

## Ethics statement

Ethical review and approval was not required for the study on human participants in accordance with the local legislation and institutional requirements. Written informed consent from the patients/participants or patients/participants' legal guardian/next of kin was not required to participate in this study in accordance with the national legislation and the institutional requirements.

## Author contributions

MH: Conceptualization, Data curation, Formal analysis, Writing – original draft. YL: Conceptualization, Methodology, Software, Visualization, Writing – review & editing. YC: Methodology, Writing – review & editing. WD: Project administration, Supervision, Methodology, Writing – review & editing.

## Footnotes

^1^ https://www.finngen.fi/en
